# Fish Paralog Proteins RNASEK-a and -b Enhance Type I Interferon Secretion and Promote Apoptosis

**DOI:** 10.3389/fimmu.2021.762162

**Published:** 2021-11-22

**Authors:** Zhi-Chao Sun, Zeyin Jiang, Xiaowen Xu, Meifeng Li, Qing Zeng, Ying Zhu, Shanghong Wang, Yuanyuan Li, Xiao-Li Tian, Chengyu Hu

**Affiliations:** ^1^ College of Life Science, Nanchang University, Nanchang, China; ^2^ Human Aging Research Institute, Nanchang University, Nanchang, China; ^3^ Jiangxi Key Laboratory of Human Aging, Nanchang University, Nanchang, China; ^4^ Blood Transfusion Department, First Affiliated Hospital of Gannan Medical University, Ganzhou, China; ^5^ Department of Gastroenterology, The First Affiliated Hospital of Nanchang University, Nanchang, China

**Keywords:** RNASEK-a, RNASEK-b, *Ctenopharyngodon idella*, type I interferon, apoptosis

## Abstract

Type I interferon and apoptosis elicit multifaceted effects on host defense and various diseases, such as viral infections and cancers. However, the gene/protein network regulating type I interferon and apoptosis has not been elucidated completely. In this study, we selected grass carp (*Ctenopharyngodon idella*) as an experimental model to investigate the modulation of RNASEK on the secretion of type I interferon and apoptosis. We first cloned two paralogs RNASEK-a and -b in grass carp, defined three exons in each gene, and found the length of both coding regions is 306 bp with 73.27% of protein homology. The protein sequences of the two paralogs are highly conserved across species. Two proteins were mainly localized in early and late endosomes and endoplasmic reticulum. Further, quantitative real-time PCR demonstrated that dsRNA poly I:C and grass carp reovirus upregulated RNASEK-a and -b in grass carp cells and tissues. Overexpression of RNASEK-a and -b individually induced type I interferon expression and the phosphorylation of IRF3/IRF7 shown by Western blot and immunofluorescent staining, increased *Bax*/*Bcl-2* mRNA ratio, DNA fragmentations, TUNEL-positive cells, and the proportion of Annexin V-positive signals in flow cytometry, and activated eIF2α, opposite to that observed when RNASEK-a and -b were knocked down in multiple cell types. Taken together, we claim for the first time that fish paralog proteins RNASEK-a and -b enhance type I interferon secretion and promote apoptosis, which may be involved in the phosphorylation of IRF3/IRF7 and eIF2α, respectively. Our study reveals a previously unrecognized role of RNASEK as a new positive regulator of type I interferon and apoptosis.

## Highlights

1. Grass carp RNASEK-a and -b can be upregulated by dsRNA poly I:C and grass carp reovirus.
2. Grass carp RNASEK-a and -b mainly localize in endosomes and endoplasmic reticulum but rarely in mitochondria and lysosomes.
3. Grass carp RNASEK-a and -b individually enhance type I interferon secretion and promote apoptosis.

## Introduction

RNASEK, also named as RNase κ or RNase K, belongs to a previously identified family. RNASEK is conserved in nearly all metazoans and widely expressed in all tissues and developmental stages (1–3). It was discovered for the first time as a novel endoribonuclease with the ability to cleave the 5′ region of *ompA* transcripts in *Escherichia coli* (4). In the early phase, RNASEK is recognized as a proteolytic fragment of RNase E because these two proteins cleave *ompA* mRNA at an identical site known to be rate-determining for degradation (5). *Ceratitis capitata* RNase (Cc RNase), one member of the RNASEK family, was first cloned and exhibited ribonucleolytic activity against poly(C) and poly(U) synthetic substrates and rRNA (1). Further gene structure analysis showed that the genome of RNASEK consists of three exons with a highly conserved size interrupted by two introns with various lengths across almost all taxa (3). Human RNASEK encoded by a single-copy gene was subsequently cloned and shares 100% amino acid identity with a large number of mammalian counterparts such as monkeys, mice, and rats. Purified human RNASEK preferentially cleaves ApU and ApG phosphodiester bonds, the rate of which correlates with multiple conditions such as enzyme concentration, pH, and ions (2). In addition, the intramolecular disulfide bond formed by cysteine residues 6 and 69 is required for the catalytic activity of human RNASEK (6). In short, the characterization of high conservation, wide expression, and endonuclease activity of RNASEK family members suggest that they play a pivotal role in biological processes.

The RNASEK family has been reported to be involved in multiple immune-related diseases, including cancers and viral infections (7–11). For instance, to search for novel therapeutic targets for breast and ovarian cancers, Gkratsou et al. (7) treated both cell lines with paclitaxel, an anti-cancer drug exerting its anti-cancer function by inducing cell apoptosis, and observed that the *RNASEK* mRNA level was upregulated, suggesting that RNASEK may participate in apoptosis-related pathways. The other study reported that RNASEK overexpression is associated with the decreased risk of prostate cancer (CaP) development, less advanced and less aggressive tumors, longer progression-free survival, and favorable prognosis in benign prostate hyperplasia and CaP patients (8).

A number of studies present that RNASEK is associated with the internalization of multiple viruses, including human rhinovirus, influenza A virus, and dengue virus (9–11), and the loss of RNASEK results in enlarged clathrin-coated pits at the cell surface and increases endo-lysosomal acidity, to some extent, preventing the entry and replication of various viruses that enter cells from endosomal compartments but not at the plasma membrane (9). Furthermore, it has been demonstrated that the requirement of RNASEK for viral entry appears specific, evidenced by the following: a) RNASEK is required for viral uptake rather than attachment to cell surface (10), b) RNASEK assists in the internalization of diverse acid-dependent viruses which enter cells mainly through the clathrin-dependent pathway (10), and c) RNASEK modulates viral uptake by a size-dependent pathway (11). In addition, Hackett et al. (11) found that RNASEK positively regulates LY6E-dependent tubule formation required for early events of flavivirus endocytosis; on the contrary, loss of RNASEK attenuated LY6E tubularization-mediated WNV-Kunjin infection. Altogether, RNASEK is essential for cancer progress and viral infections, suggesting that it may be involved in innate immune response. However, the biological role RNASEK plays in host defense remains to be elucidated.

Given the fact that RNASEK participates in multiple immune-related pathological processes, we propose our hypothesis that RNASEK plays an important role in immunoresponses. Currently, two RNASEK paralogs, RNASEK-a and -b, were deposited in a plethora of teleost fish such as *Danio rerio*, *Carassius auratus*, and *Sinocyclocheilus rhinocerous* (https://www.ncbi.nlm.nih.gov/), but the roles they play in fish innate immune system are unclear.

As a group of lower vertebrates, fish innate immune system is required for host defense against viral infections. Recently, accumulating pieces of evidence show that gene/protein network regulating innate immunity is relatively conservative between fish and mammals, such as type I interferons (IFNs), pattern recognition receptors (PRRs), and IFN regulatory factors (IRFs) (12–15). Fish type I IFNs have been classified into group I and group II and further into subgroups a, d, e, and h and b, c, and f, respectively (15, 16). PRR-induced secretion of type I IFNs is known as the first line for fish to eliminate invading viruses. As already known, Toll-like receptors (TLRs) and retinoic acid-inducible gene I (RIG-I)-like receptors (RLRs) are two important PRRs (15). TLRs are type I integral membrane proteins with an ectodomain of leucine-rich repeat motifs binding ssRNA, dsRNA, or CpG DNA, then recruit appropriate adaptors (e.g., TRIF/TICAM-1, MyD88, or TIRAP) to their cytosolic Toll-IL-1 receptor (TIR) domains, thereby interacting with TANK binding kinase 1 (TBK1) to activate IRF3/IRF7 for induction of type I IFNs (15, 17–19). RIG-I and MDA5, two common RLRs, detect cytosolic ssRNA or dsRNA, and then interact with the mitochondrial adaptor MAVS (also called IPS-1) to trigger the phosphorylation of IRF3/IRF7 for enhancing type I IFN secretion (15, 17, 20, 21). Although plenty of efforts have been made on clarifying the molecular mechanism of fish type I IFN system, whether fish RNASEK-a and -b modulate type I IFN secretion remains elusive.

Apoptosis, a form of programmed cell death, is essential for maintaining organismal homeostasis and plays key roles in innate immunity and cytotoxicity. In fish, the gene/protein network modulating apoptosis has been investigated in multiple toxicological experiments and viral diseases, which are, to some extent, homologous to the mammalian counterpart (22–24). In grass carp hepatocytes (L8824) challenged by chlorpyrifos, oxidative stress was sharply enhanced, inducing the expression of PTEN, thereby negatively regulating the PI3K/AKT pathway to promote apoptosis (23). Mao et al. (24) reported that Fas/FasL signaling positively regulated nervous necrosis virus-induced apoptosis in Pacific cod and EPC cells. Altogether, a plethora of molecules has been evidenced to participate in apoptotic signaling pathways, but whether fish RNASEK-a and -b regulate apoptosis is unknown.

Grass carp (*Ctenopharyngodon idella*), an important aquaculture species, has attracted a lot of research on its immune system. Up to now, four grass carp type I IFNs have been uncovered, including Ci-IFN1 (DQ357216) and Ci-IFN2-4 (KU182641–KU182643) (15). In the present study, we used grass carp as a model to investigate the regulation of RNASEK on type I IFN (DQ357216) secretion and apoptosis, two responses required for innate immunity. In detail, we cloned the coding sequence (CDS) and genome of grass carp *RNASEK-a* and *-b* and found that both of them can induce IRF3/IRF7-mediated type I IFN production and eIF2α-dependent apoptosis in multiple grass carp cells.

## Material and Methods

### Fish and Cell Lines

Grass carp (10–20 g, Nanchang Shenlong Fisheries Development Co., Ltd., Jiangxi, China) were acclimatized in a specific pathogen-free animal tank for at least 2 weeks at 28°C prior to the experiments. For each experiment, three different fishes were killed to obtain the mixed sample.

Three stable cell lines, *C. idella* kidney (CIK), ovary (CO), and liver (CL, L8824) cells, were used in this study. CIK and CO cells were a gift from Professor Pin Nie (Institute of Hydrobiology, Chinese Academy of Sciences). CL cells were provided by Professor Jinnian Li (Anhui Agricultural University, China). Keeping in view CO cells were sensitive to the alteration of pH but CIK and CL cells were not, they were grown in different conditions. CIK (25) and CL (26) cells were cultured in Medium 199 (Corning, USA) containing 10% fetal bovine serum (FBS; Gibco, USA) and 0.6% penicillin–streptomycin liquid (Beijing Solarbio Science & Technology Co., Ltd., China) at 28°C. CO (25) cells were grown in the same condition as that of CIK cells except for the addition of 5% CO_2_. These cell lines were used for functional and confocal assays.

### Cloning and Sequence Analysis of Grass Carp RNASEK-a and -b

Genomic DNA and total RNA were extracted from grass carp tissues (50 mg for each tissue) by using TaKaRa MiniBEST Universal Genomic DNA Extraction Kit Ver.5.0 (Takara Bio, China) and RNA simple Total RNA Kit (Tiangen Biotech, China), respectively. The RNA sample was synthesized into cDNA by use of the PrimeScript RT reagent Kit with gDNA Eraser Perfect Real Time (Takara Bio, China). Homologous primers RNASEK-a-ORF-F/R and -b-ORF-F/R were designed according to the CDS of *Carassius auratus* RNASEK-a (XM_026267089) and RNASEK-b (XM_026258901). The above cDNA templates were used to perform polymerase chain reaction (PCR) with specific primers, the programs of which were 95°C for 3 min followed by 35 cycles of 95°C for 30 s, 55°C for 30 s, and 72°C for 30 s, and then a final elongation at 72°C for 10 min. The PCR products were observed through agarose gel electrophoresis. Target DNA fragments were recycled from agarose gels, then cloned into pEASY-T1 vectors (Takara Bio, China), and finally confirmed by DNA sequencing (Tsingke, Beijing, China). Genomic DNA was subjected to the same cloning method as that of total RNA in the absence of reverse transcription. Thus, the CDS region and genome of both RNASEK-a and -b were obtained. The genomic structure of RNASEK from various taxa was compared. Sequence similarity analysis was conducted by Nucleotide BLAST of NCBI (http://www.ncbi.nlm.nih.gov/blast). Protein domains were predicted by the SMART program (http://smart.embl-heidelberg.de/smart/set_mode.cgi?NORMAL=1). According to the amino acid sequences of the RNASEK family, multiple sequence alignment was performed *via* BioEdit software, and the phylogenetic tree was constructed by using the neighbor-joining algorithm from MEGA6.0 program. The primers used are listed in **Table S1**. GenBank accession numbers of selected RNASEK orthologs from various species are listed in **Table S2**.

### Plasmids and Recombinant Construction

PcDNA3.1, p3×FLAG-Myc-CMV™-24, pEGFP-C1, pDsRed2-C1, pGPU6/Neo, and PLKO-U6-PGK-mCherry (RFP)-2A-NEO (G418) plasmids were purchased from Invitrogen (USA), Transgen (Beijing, China), Promega (USA), BioVector (Chongqing, China), GenePharma (Suzhou, China), and Tsingke (Beijing, China), respectively. The CDS regions of grass carp RNASEK-a and -b were separately inserted into pcNDA3.1, pEGFP-C1, and p3×FLAG-Myc-CMV™-24 vectors to construct recombinant plasmids. Additionally, grass carp TBK1 CDS (MH545564.1) was used to obtain pcDNA3.1-TBK1 recombinant. The CDS regions of grass carp Rab5 (MF598473.1) and Rab7 (MF598474.1) were individually constructed into pDsRed2-C1 vectors (finished by Sangon Biotech, China). Subsequently, short hairpin RNA (shRNA) was designed. The shNC and shRNASEK-b-22 were cloned into pGPU6/Neo plasmids (named GP-shNC and GP-shRNASEK-b-22) as well as another shNC, shRNASEK-b-120, and shRNASEK-b-226 were inserted into PLKO-U6-PGK-mCherry (RFP)-2A-NEO (G418) vectors. The constructed recombinants pcDNA3.1-RNASEK-a/b, pEGFP-RNASEK-a/b, and pcDNA3.1-TBK1 were used for overexpression experiments. The recombinant plasmids pEGFP-RNASEK-a/b, p3×FLAG-RNASEK-a/b, and pDsRed2-Rab5/7 were utilized for subcellular localization assays. The shRNA recombinants GP-shNC, GP-shRNASEK-b-22, shNC, shRNASEK-b-120, and shRNASEK-b-226 were used to perform knockdown of RNASEK-b in target cells. All recombinant plasmids were confirmed *via* DNA sequencing (Tsingke, Beijing, China). The primers and shRNA used are listed in **Table S1**.

### Cell Transfection

Cells were seeded to six-well culture plates (Guangzhou Jet Bio-Filtration Co., Ltd., China) and 0.17 mm glass-bottom culture dishes (Wuxi NEST Biotechnology Co., Ltd., China) to grow. When reaching 60%–80% of confluence, cells were transfected with 2 or 1.5 μg of recombinant plasmids with 4 or 3 μl Lipofectamine 2000 (Invitrogen, USA, 11668019) or Lipo8000™ (Beyotime Biotechnology, China) Transfection Reagent for each sample, respectively. For the knockdown experiments, the negative control RNA oligonucleotides (termed NC) and specific siRNA against RNASEK-a-22 and -101 (termed siRNASEK-a-22 and -101) were designed by Suzhou GenePharma (**Table S1**). When cells grew to 60%–80% confluence on six-well plates, 5 μl of 20 μmol/L siRNA was transfected using an equal volume of Lipofectamine 2000 or Lipo8000™ Transfection Reagent. Twenty-four hours post-transfection, transcriptional expression analysis, immunofluorescent staining, and subcellular localization were performed. Thirty-six hours after transfection, apoptosis-related assays and Western blot were conducted. The transfection methods were according to the instructions of the manufacturer. The siRNA used is listed in **Table S1**.

### Subcellular Localization and Immunofluorescence

CIK cells were used for subcellular localization and immunofluorescence. Cells were seeded to 0.17 mm glass-bottom culture dishes. When reaching 60%–80% of confluence, cells were transfected with pEGFP-RNASEK-a or -b; to investigate whether RNASEK-a/b localizes to endosomes, pEGFP-RNASEK-a and -b were individually co-transfected with the endosome markers pDsRed-Rab5/7. Twenty-four hours post-transfection, cells were fixed with 4% paraformaldehyde (Bio-Medical Assay Co., Ltd., China) for 10 min and dyed with 2-(4-amidinophenyl)-6-indolecarbamidine dihydrochloride (DAPI, 10 μg/ml; Sangon Biotech, China) for 20 min in darkness. In addition, MitoTracker^®^ Red CMXRos (Cell Signaling Technology, USA, 9082S), Lyso-Tracker Red (Beyotime Biotechnology, China, C1046), and ER-Tracker Red (Beyotime Biotechnology, China, C1041) were used to trace the mitochondria, lysosomes, and endoplasmic reticulum after cells were transfected with pEGFP-RNASEK-a or -b to observe the relationships between each other, the protocols of which were based on the instructions of the manufacturer. To examine whether RNASEK-a is colocalized with RNASEK-b, cells were transfected with p3×FLAG-RNASEK-a and pEGFP-RNASEK-b together. After 24 h of transfection, cells were fixed as above and permeabilized with 0.5% Triton X-100, then blocked with 5% bovine serum albumin, followed by incubation with the FLAG-tag antibody (1:5,000, Sigma, USA, F1804) (27) for 12 h (overnight) at 4°C. The next day, cells were incubated in the secondary antibody goat anti-mouse Cy3 for 1 h (1:200, BioLegend, USA, 405309) and counterstained with DAPI for 20 min without light. To assess the alteration of type I IFN protein expression, immunofluorescence was performed with type I IFN antibody (1:200, saved in our lab) (28) as that of FLAG-tag, in which the secondary antibody was substituted with donkey anti-rabbit Cy3 (1:200, BioLegend, USA, 406402). All finished preparations were photographed under 63× oil immersion objective lens (630× magnification) or 200× magnification of the Zeiss confocal microscope (confocal microscope LSM800, Zeiss, Germany) and analyzed by the Zen software (Zeiss, Germany).

### Expression Profiles of Grass Carp RNASEK-a and -b

Healthy grass carp were intraperitoneally injected with poly I:C (5 mg/kg, Sigma, USA) or 100 μl of live grass carp reovirus-097 (GCRV-097, 1 × 10^−8^ TCID_50_; provided by Jian-Guo Su lab of Huazhong Agricultural University). Total RNA of each tissue (50 mg) was extracted from the brain, eye, intestine, gill, skin, spleen, liver, and kidney of grass carp at 6, 12, 24, 48, and 72 h post-stimulation, then cDNA was prepared as above. For the poly I:C group, only 6, 12, and 24 h were included because in our work we observed the expression of *RNASEK-a* and *-b* elevated after poly I:C stimulation and then declined almost to basal levels in most tested tissues, suggesting these two genes/proteins generated an early response to poly I:C *in vivo*.

CIK cells were seeded on six-well culture plates, then transfected with 5 μl poly I:C (1 mg/ml) or infected with 50 μl live GCRV-097 (1 × 10^−8^ TCID_50_) using 5 μl Lipofectamine 2000 Transfection Reagent. Total RNA of 1 × 10^6^ cells was extracted at 0 h (without treatments) and 6, 12, 24, 48, and 72 h post-stimulation for each group. The obtained RNA was used to synthesize cDNA as above.

The prepared cDNA templates were utilized to perform expression analysis of *RNASEK-a* and *-b* in grass carp tissues and CIK cells by using quantitative real-time PCR (qRT-PCR). qRT-PCR was carried out in triplicate for each template with TB Green Premix Ex Taq II (TaKaRa Bio, China) on CFX Connect™ (Bio-Rad, USA). β-Actin was used as an internal control. All data were calculated with the 2^−ΔΔCT^ method to obtain relative normalized expression of genes, such as *RNASEK-a*, *RNASEK-b*, *type I IFN*, *Bcl-2*, and *Bax*. The primers used are listed in **Table S1**.

### Western Blot

CL or CO cells were inoculated in six-well culture plates to grow to 60%–80% of confluence, then transfected with expression plasmids and shRNA for overexpression and knockdown of target genes, respectively. Thirty-six hours post-transfection, cells were lysed in 100 μl of NP40 lysis buffer (Beyotime Biotechnology, China) with the addition of 1 mmol/L phenylmethyl sulfonyl fluoride (PMSF, Sangon Biotech, China), 1 μg/ml leupeptin (Sangon Biotech, China), 1 μg/ml aprotinin (Sangon Biotech, China), and 1 mmol/L phosphate inhibitor cocktail (CWBIO, Beijing, China). Cell lysates were aspirated into a 1.5-ml tube and subjected to centrifugation at 12,000 r/min for 10 min at 4°C to remove cell debris. The acquired supernatant was boiled together with a quarter volume of 5× SDS-loading buffer containing 3.5% β-mercaptoethanol at 95°C for 10 min. The protein samples were separated through SDS-polyacrylamide gel electrophoresis (PAGE) and then transferred to nitrocellulose membranes (Millipore, USA). The membranes were blocked with 5% non-fat milk for 1 h and incubated in the specific antibodies for 12 h (overnight) at 4°C. The next day, the membranes were incubated in horseradish peroxidase-conjugated secondary antibody for 1 h at a regular temperature. Finally, the membranes were exposed using a chemiluminescence imaging system (CLINX, China). Specific antibodies used here are against grass carp type I IFN (1:800, saved in our lab) (28), phosphorylated IRF3 (p-IRF3; 1:1,000, Beyotime Biotechnology, China, AF1594) (27), basal and phosphorylated IRF7 (p-IRF7; 1:1,000, HuaBio, China, ET1610-89) (29), eIF2α (1:500, saved in our lab) (30), phosphorylated eIF2α (p-eIF2α; 1:1,000, Abcam, UK, ab32157) (30), and GAPDH (loading control; 1:10,000, saved in our lab) (30). All commercial antibodies against p-IRF3, IRF7/p-IRF7, and p-eIF2α cross-react with grass carp, which is confirmed by transfection of pEGFP-IRF3, -IRF7, and -eIF2α into grass carp cells followed by comparison of the size of the basal and GFP recombinant proteins of each gene in our previous work (27, 29, 30).

### Cell Apoptosis

CIK, CL, or CO cells were used to test cell apoptosis *via* four methods, namely, qRT-PCR, DNA ladder (DNA fragmentations of whole multiples of 180–200 bp), TdT-mediated dUTP nick-end labeling (TUNEL), or flow cytometry. CIK, CL, and CO cells were inoculated in six-well culture plates to reach 60%–80% of confluence, then transfected with recombinant plasmids or siRNA/shRNA for 36 h. Total RNA was extracted from CIK and CL cells to perform qRT-PCR. The ratio of *Bax* to *Bcl-2* mRNA expression (*Bax*/*Bcl-2* mRNA ratio) was used to assess cell apoptosis. Total DNA was extracted from CIK and CL cells and separated by agarose gel electrophoresis to observe the DNA ladder which reflects the degree of cell apoptosis. In addition, CIK cells were subjected to TUNEL staining by using an inverted fluorescence microscope (Zeiss, Germany) and One-Step TUNEL Apoptosis Assay Kit (Beyotime Biotechnology, China, C1086) according to the instructions of the manufacturer, in which apoptotic cells are marked by TUNEL-positive green signals. For CO cells, the degree of apoptosis was determined by a flow cytometer (FACSVerse flow cytometer, BD, USA) and Annexin V, FITC Apoptosis Detection Kit (Dojindo, Japan, AD10), the protocols of which were based on the instructions of the manufacturer. Subsequently, the proportion of Annexin V-positive cells was analyzed for each sample through FlowJo vX.0.7 software.

### Statistical Analysis

All values were analyzed as mean ± SD. Statistical analysis was performed by the two-sample *t*-test and shown with figures produced by GraphPad Prism Version 8. The significance level used was *P <*0.05.

## Results

### CDS Analysis of Grass Carp RNASEK-a and -b

For both grass carp RNASEK-a and -b, the CDS consists of 306 bp encoding a polypeptide of 101 amino acids with two transmembrane domains and four Cys residues (**Figure S1A**). To investigate the conservation of the RNASEK family members, multiple sequence alignment was conducted according to their amino acid sequences. Both grass carp RNASEK-a and -b are highly conserved across 10 selected species, consisting of 8 mammals and 2 fish, especially with the matched genes from zebrafish (**Figure S1B**). For further analysis of the evolutionary status of RNASEK family members, the phylogenetic tree was constructed. As shown in **Figure S2**, RNASEK exists in nearly all metazoans. Mammalian ones are extremely consistent with each other. RNASEK-a and -b paralogs are distinguished only in bony fish. RNASEK-b orthologs are located on only one branch, displaying a closer genetic relationship than RNASEK-a ones which are divided into three different branches. Taken together, the RNASEK family is conservative from invertebrates to mammals, suggesting that it plays a key role in biological processes.

### Genomic Structure of RNASEK Family Genes From Various Taxa

The comparison of *RNASEK* gene structure was performed previously (3). However, in recent years, a plethora of *RNASEK* family genes were newly discovered in various taxa, including fish *RNASEK-a* and *-b.* To understand the gene structure of the *RNASEK* family more completely, we searched for *RNASEK* homologs from 22 animal species (including mammals, bony fish, birds, amphibians, turtles, and arthropods) by using NCBI (https://www.ncbi.nlm.nih.gov/) to compare their intron/exon structure. In general, the genome of *RNASEK* is composed of three exons with the exception of two exons for *RNASEK* of *Phasianus colchicus* and *Chelonoidis abingdonii* as well as four exons for *RNASEK-a* of *Seriola dumerili*, *Monopterus albus*, and *Micropterus salmoides*. The length of the *RNASEK* genome is highly divergent, ranging from 421 to 12,730 bp, and the reason for this is a higher degree of variability in introns than exons. From primitive animals to human, the size, positions, and numbers of exons are relatively conserved, of which exons 1, 2, and 3 individually contain 78, 77, and 142 bp for all examined mammalian *RNASEK* and 78, 83, and 145 bp for all listed fish *RNASEK-b* and one half of the fish *RNASEK-a*. As for the remaining three fish *RNASEK-a*, exons 1, 2, 3, and 4 individually contain 75, 80, 124–127, and 18 bp, respectively. Other animal *RNASEK* genomes have two or three exons with high conservation in the same taxon, and in particular, the size of exon 1 is highly conserved in all listed species (ranging from 69 to 85 bp) (**Figure S3**). However, the length of intronic regions of the *RNASEK* genome varies much from 66 to 11,781 bp, which is related to taxonomic groups. For instance, there is a slight difference from each other in mammalian ones, from 250 to 3,714 bp in fish *RNASEK-b*, from 102 to 466 bp in fish *RNASEK-a* that consisted of three exons, from 494 to 3,650 bp in another half of fish *RNASEK-a*, approximately 66 bp in *Drosophila melanogaster RNASEK*, and from 649 to 11,781 bp in that of *Petromyzon marinus* (**Figure S3**). Thus, the variations of *RNASEK* homolog genes, to a large degree, are attributed to the differences in their intronic architecture. Additionally, fish *RNASEK-a* has two forms of composition but only one for *RNASEK-b*, which is coincident with the results from the above phylogenetic tree.

### Poly I:C and GCRV Upregulate RNASEK-a and -b Expression

**Figure 1:**
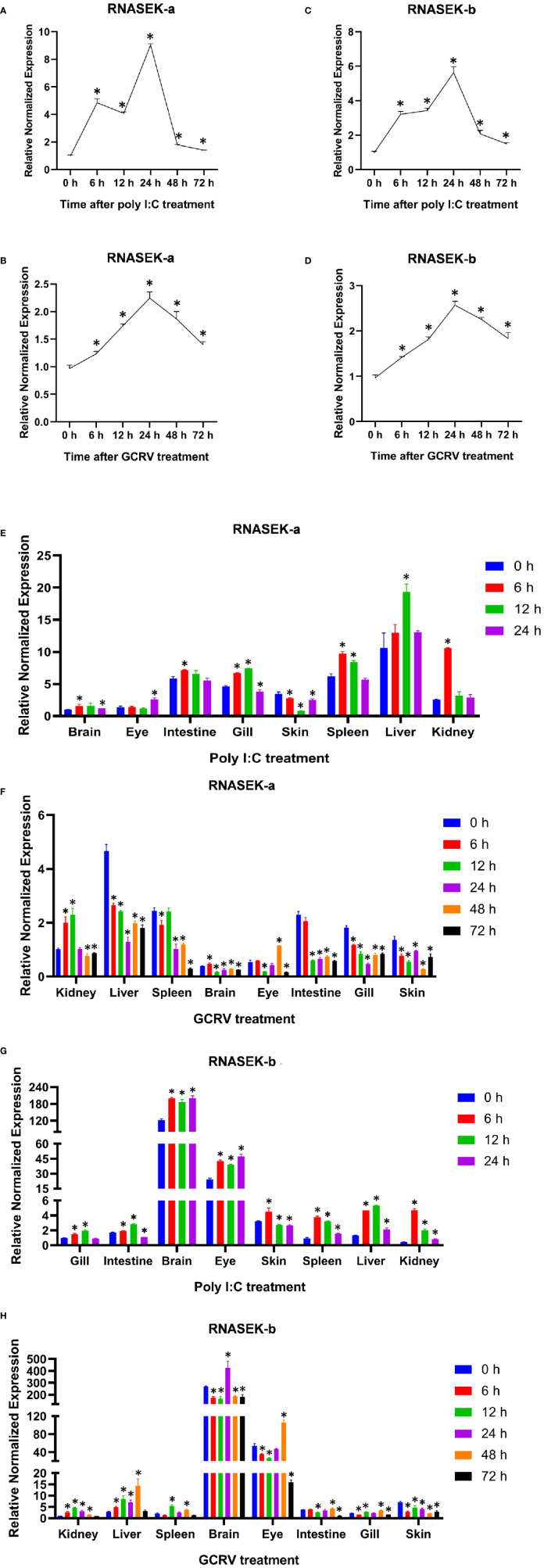
Expression analysis of *RNASEK-a* and *-b* in CIK cells and grass carp tissues. **(A, B)** The relative normalized mRNA expression of *RNASEK-a* in CIK cells exposed to the poly I:C **(A)** and GCRV **(B)** stimulation for indicated time periods. **(C, D)** The mRNA levels of *RNASEK-b* were determined in CIK cells treated with the same way as that of *RNASEK-a*. **(E, F)** The change folds of *RNASEK-a* mRNA in different grass carp tissues (brain, eye, intestine, gill, skin, spleen, liver, and kidney) injected with poly I:C **(E)** and GCRV **(F)** for multiple time periods. **(G, H)** The mRNA expression of *RNASEK-b* was tested in grass carp tissues challenged by the same stimuli as that of *RNASEK-a*. β-Actin was used as an internal control. The mRNA expression of genes was normalized to that of β-actin to obtain their relative normalized expression by using the 2^−ΔΔCT^ method **(A–H)**. The mRNA expression of *RNASEK-a* in the brain **(E)** and kidney **(F)** at 0 h was normalized to 1, and the same for that of *RNASEK-b* in the gill **(G)** and kidney **(H)**. The controls are 0 h for each group **(A–H)**. The values are mean ± SD. Each sample was run by qRT-PCR in triplicate. These results are representative of three independent experiments. *P < 0.05 versus the relative controls.

To assess the expression profiles of grass carp *RNASEK-a* and *-b via* qRT-PCR in CIK cells, we cultured them in the context of stimulation of poly I:C and GCRV-097 for the indicated time periods (0, 6, 12, 24, 48, and 72 h). Both *RNASEK-a* and *-b* transcripts were significantly elevated after each stimulation and peaked at 24 h (up to 2.25–9.04-fold versus 0 h), and then declined (**Figures 1A–D**), the trends of which were similar with those of *type I IFN* (**Figure S4**).

To perform the expression analysis of *RNASEK-a* and *-b* in grass carp tissues by qRT-PCR, healthy grass carp were intraperitoneally injected with poly I:C and GCRV-097 for various time periods (0, 6, 12, 24, 48, or 72h). *RNASEK-a* mRNA expression was the highest in the liver and nearly the lowest in the brain and eye for both stimulation (**Figures 1E, F**). In the poly I:C group, *RNASEK-a* transcripts increased after treatments except for the skin and almost peaked at 6, 12, or 24 h for different tissues (**Figure 1E**). In the GCRV group, *RNASEK-a* mRNA expression was upregulated post-stimulation in the kidney, brain, and eye and peaked at 12, 6, and 48 h, respectively (**Figure 1F**). The expression patterns of *RNASEK-b* were investigated in the same way as those of *RNASEK-a*. *RNASEK-b* expression reached the highest in the brain and very low in the kidney, spleen, intestine, gill, and skin. In the poly I:C and GCRV groups, the transcriptional levels of *RNASEK-b* elevated post-treatments apart from the skin (GCRV group), which peaked at 6, 12, 24, or 48 h in different tissues (**Figures 1G, H**). Besides, both genes were widely expressed in all tested tissues and their expression levels declined from the peak (**Figures 1E–H**).

In summary, dsRNA poly I:C and GCRV can upregulate *RNASEK-a* and *-b* expression *in vitro* and *in vivo*.

### Grass Carp RNASEK-a and -b in Part Localize to Endosomes and Endoplasmic Reticulum

**Figure 2:**
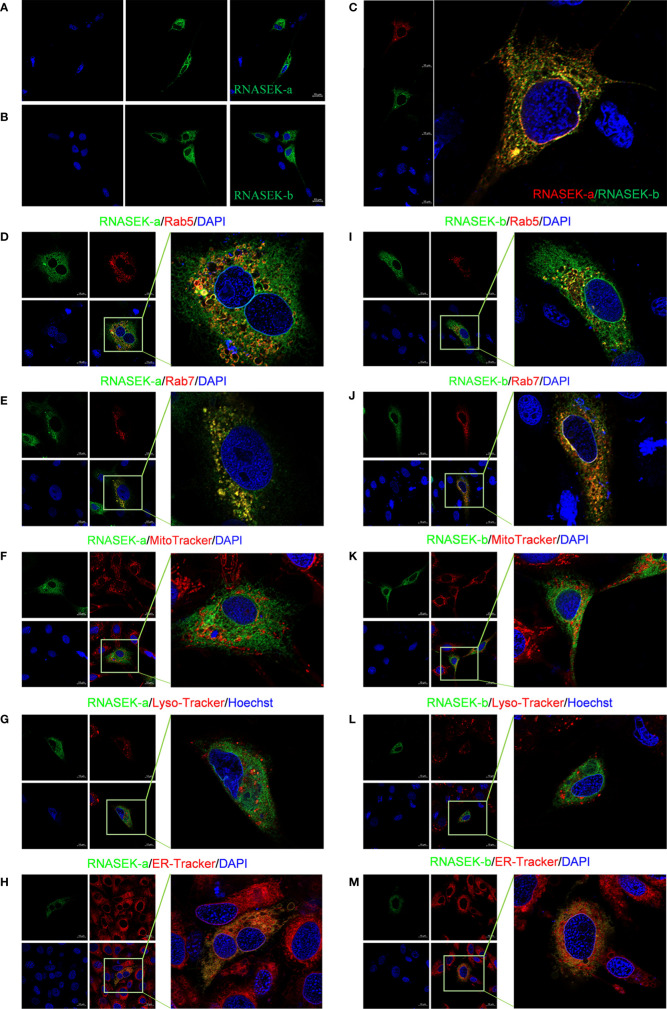
Subcellular localization of RNASEK-a and -b within CIK cells. **(A, B)** Both GFP-tags of RNASEK-a **(A)** and -b **(B)** were exclusively expressed in the cytoplasm (green, GFP; blue, DAPI). **(C)** RNASEK-a and -b were colocalized with each other (red, p3×Flag-RNASEK-a; green, pEGFP-RNASEK-b; blue, DAPI; yellow, colocalization). **(D, E)** RNASEK-a was colocalized with early endosome marker Rab5 **(D)** and late endosome marker Rab7 **(E)** in the cytoplasm (green, pEGFP-RNASEK-a; red, pDsRed-Rab5 and -Rab7; blue, DAPI; yellow, colocalization). **(F, G)** RNASEK-a was rarely colocalized with the mitochondria **(F)** and lysosomes **(G)** in the cells (green, pEGFP-RNASEK-a; red, mitochondria indicated by MitoTracker and lysosomes traced by Lyso-Tracker; blue, DAPI or Hoechst; yellow, colocalization). **(H)** RNASEK-a was in part localized to the endoplasmic reticulum (green, pEGFP-RNASEK-a; red, endoplasmic reticulum traced by ER-Tracker; blue, DAPI; yellow, colocalization). **(I–M)** Subcellular localization of RNASEK-b was the same as that of RNASEK-a **(D–H)**. Experiments were performed by transfection of only one kind of recombinant plasmid for one protein localization or two different ones for colocalization. The results are representative of three independent experiments. Images were taken under 63× oil immersion objective lens (630× magnification) by using the Zeiss confocal microscope. Scale bar, 10 μm.

To dissect the function of grass carp RNASEK-a and -b, we performed subcellular localization of them in CIK cells. We first traced the RNASEK-a and -b through separately overexpressing the recombinants pEGFP-RNASEK-a and -b in CIK cells. As shown in **Figures 2A, B**, RNASEK-a and -b (green) existed only in the cytoplasm. Afterward, we expressed p3×FLAG-RNASEK-a (red) and pEGFP-RNASEK-b (green) together and observed that they were colocalized with each other (yellow) in the cytoplasm (**Figure 2C**). To investigate the fine localization of RNASEK-a and -b, pEGFP-RNASEK-a and -b were individually transferred into the cells together with the recombinants pDsRed2-Rab5 (early endosomal marker, red) or -Rab7 (late endosomal marker, red). Interestingly, both RNASEK-a and -b had colocalization (yellow) with these two endosome markers (**Figures 2D, E, I, J**). Furthermore, CIK cells were stained with MitoTracker (red), Lyso-Tracker (red), and ER-Tracker (red) after transfection of pEGFP-RNASEK-a and -b, demonstrating that both RNASEK-a and -b mainly localized to the endoplasmic reticulum but rarely to the mitochondria and lysosomes (**Figures 2F–H, K–M**). In brief, grass carp RNASEK-a and -b have similar subcellular localization, which is localized mainly to the endosomes and endoplasmic reticulum but rarely to the mitochondria and lysosomes in the cytoplasm.

### Grass Carp RNASEK-a and -b Promote Type I IFN Expression

**Figure 3:**
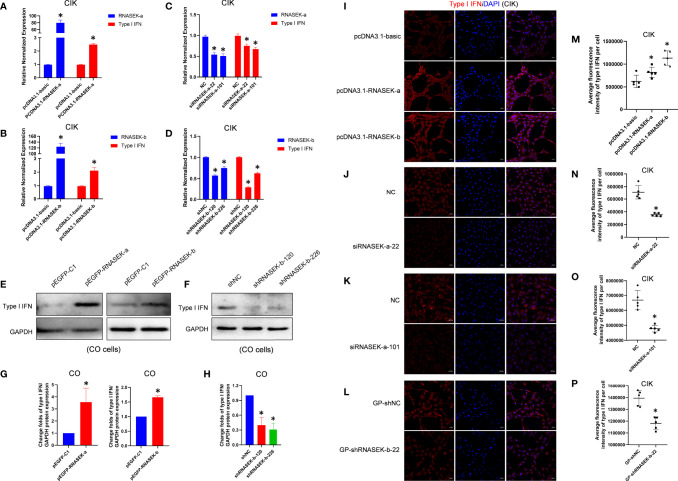
Grass carp RNASEK-a and -b individually promote type I IFN expression. **(A–D)** In CIK cells, *type I IFN* mRNA expression was assessed after overexpression of RNASEK-a **(A)** and -b **(B)** as well as knockdown of RNASEK-a **(C)** and -b **(D)** by using qRT-PCR in triplicate. **(E, F)** In CO cells, type I IFN protein expression was examined after overexpression of RNASEK-a and -b **(E)** as well as knockdown of RNASEK-b **(F)** through Western blot with GAPDH as a loading control. **(G, H)** The grayscale values of type I IFN bands representing **(E)** and **(F)** were individually calculated by the ImageJ software (*n* = 3–5; the control data were normalized to 1). **(I–L)** In CIK cells, type I IFN protein expression was observed *via* immunofluorescent staining after RNASEK-a and -b overexpression **(I)**, siRNASEK-a-22 **(J)** and -101 **(K)**-mediated knockdown of RNASEK-a, and GP-shRNASEK-b-22 **(L)**-mediated knockdown of RNASEK-b. Images were taken under 200× magnification by using the Zeiss confocal microscope (red, type I IFN; blue, DAPI; scale bar, 20 μm). **(M–P)** Five random views representing **(I–L)** were selected to determine the statistical significance, respectively. Overexpression of RNASEK-a and -b was performed *via* transfection of relative recombinant plasmids, including pcDNA3.1-RNASEK-a, pcDNA3.1-RNASEK-b, pEGFP-RNASEK-a, and pEGFP-RNASEK-b. The knockdown of RNASEK-a was conducted by siRNA against RNASEK-a-22 and -101. RNASEK-b knockdown was carried out with GP-shRNASEK-b-22, shRNASEK-b-120, and -226. Cell type is marked in each image. The relative normalized mRNA expression of genes was obtained by using the 2^−ΔΔCT^ method with β-actin as an internal control **(A–D)**. The controls consist of pcDNA3.1-basic **(A, B, I, M)**, NC **(C, J, K, N, O)**, shNC **(D, F, H)**, GP-shNC **(L, P)**, and pEGFP-C1 **(E, G)**. All values are mean ± SD. The results are representative of three independent experiments. *P < 0.05 versus the controls.

To investigate whether RNASEK-a and -b are involved in innate immune response within CIK cells, we tested their effect on *type I IFN* mRNA and protein expression *via* qRT-PCR and immunofluorescence, respectively. Overexpression experiments were performed by transfection of pcDNA3.1-RNASEK-a and -b into cells for 24 h. As shown in **Figures 3A, B**, RNASEK-a and -b overexpression individually upregulated *type I IFN* transcripts by 2.49- and 2.11-fold compared with the controls. Furthermore, RNASEK-a and -b overexpression significantly enhanced the average fluorescence intensity of type I IFN protein (red), reaching 1.32- and 1.83-fold versus the control, respectively (**Figures 3I, M**). RNASEK-a knockdown was completed by transfection of siRNA against RNASEK-a-22 and -101 into cells, which individually downregulated *type I IFN* transcripts by 1.33 and 1.49 times in comparison with the control (**Figure 3C**). For immunofluorescence, these two siRNAs separately decreased the fluorescence intensity of type I IFN by 1.99- and 1.40-fold versus the controls (**Figures 3J, K, N, O**). RNASEK-b knockdown was finished by using shRNA transfection, including GP-shRNASEK-b-22, shRNASEK-b-120, and -226. The last two shRNA-mediated knockdown of RNASEK-b individually inhibited *type I IFN* mRNA expression by 3.39 and 1.59 times than the control (**Figure 3D**). In addition, GP-shRNASEK-b-22 transfection reduced the fluorescence intensity of type I IFN by 1.20-fold in comparison with the control (**Figures 3L, P**). Collectively, both RNASEK-a and -b promote type I IFN expression at transcriptional and protein levels in CIK cells.

For further validation of the above results, CO cells were used to assess type I IFN protein through Western blot. pEGFP-RNASEK-a and -b were transfected into cells to finish overexpression. As shown in **Figures 3E, G**, RNASEK-a and -b overexpression elevated type I IFN protein by 3.56- and 1.66-fold compared with the control, respectively. Given the high expression of RNASEK-b and very low abundance for RNASEK-a in CO cells, we merely performed RNASEK-b knockdown with shRNASEK-b-120 and -226. They individually downregulated type I IFN by 2.51 and 3.22 times than the control (**Figures 3F, H**). In short, both RNASEK-a and -b promote type I IFN expression in CO cells, consistent with that in CIK cells.

### Grass Carp RNASEK-a and -b Activate IRF3 and IRF7

**Figure 4:**
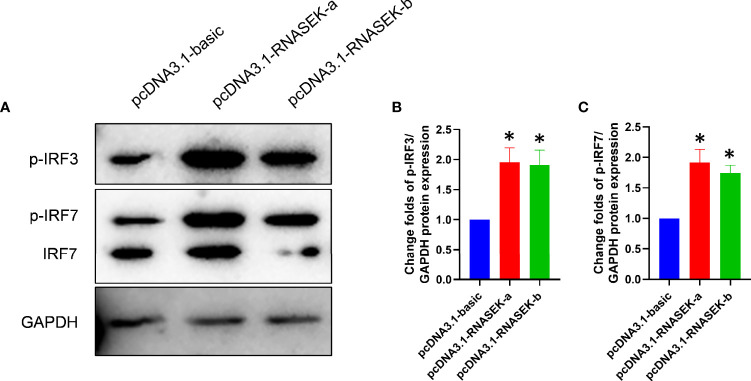
Grass carp RNASEK-a and -b individually activate IRF3 and IRF7. **(A)** In CIK cells, the protein levels of p-IRF3, p-IRF7, and IRF7 were tested by using Western blot with GAPDH as a loading control. **(B, C)** The grayscale values of p-IRF3 and p-IRF7 bands representing **(A)** were individually calculated by the ImageJ software (*n* = 3–5; the control data were normalized to 1). Overexpression of RNASEK-a and -b was conducted by transfection of pcDNA3.1-RNASEK-a and -RNASEK-b into CIK cells for 36 h, respectively. All values are mean ± SD. These results are representative of three independent experiments. *P < 0.05 versus the pcDNA3.1-basic group.

In grass carp, endosomal TLR–IRF3/IRF7 axis has been reported to induce type I IFN expression (17), which allows us to consider whether RNASEK-a and -b promote type I IFN production *via* activating IRF3/IRF7 because of their localization. For validation of this hypothesis, overexpression experiments were carried out with the transfection of pcDNA3.1-RNASEK-a and -b into CIK cells. As expected, RNASEK-a and -b overexpression individually upregulated the protein expression of p-IRF3 by 1.95- and 1.91-fold as well as p-IRF7 by 1.92 and 1.74 times versus the controls (**Figure 4**). Collectively, grass carp RNASEK-a and -b separately activate IRF3 and IRF7.

### Grass Carp RNASEK-a and -b Promote Cell Apoptosis

**Figure 5:**
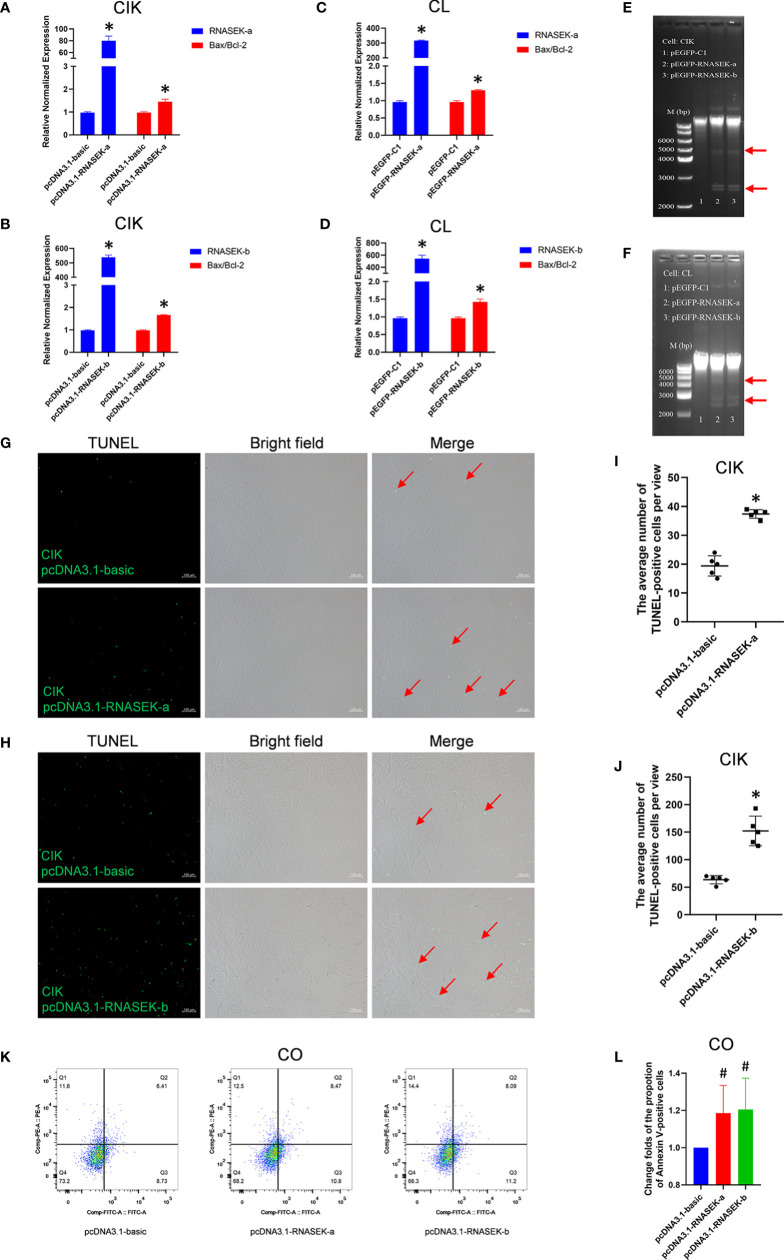
Grass carp RNASEK-a and -b individually promote cell apoptosis. **(A–D)** In CIK **(A, B)** and CL **(C, D)** cells, overexpression of RNASEK-a **(A, C)** and -b **(B, D)** individually increased *Bax/Bcl-2* mRNA ratio assessed by using qRT-PCR in triplicate. **(E, F)** Both RNASEK-a and -b overexpression increased DNA fragmentations (indicated with red arrows) shown by agarose gel electrophoresis in CIK **(E)** and CL **(F)** cells. **(G, H)** The pro-apoptosis effects of RNASEK-a **(G)** and -b **(H)** overexpression on CIK cells were assessed by TUNEL staining. Images were obtained under 100× magnification by using an inverted fluorescence microscope (green, TUNEL-positive apoptotic cells marked by red arrows; scale bar, 100 μm). **(I, J)** Five views representing **(G, H)** were randomly selected to calculate statistical significance, respectively. **(K, L)** In CO cells, the pro-apoptosis effects of both RNASEK-a and -b overexpression were evaluated by flow cytometry **(K)**, where the change folds of the proportion of apoptotic cells marked by Annexin V-FITC are shown (*n* = 3; the control data were normalized to 1) **(L)**. Overexpression of RNASEK-a and -b was performed *via* transfection of relative recombinant plasmids, including pcDNA3.1-RNASEK-a, pcDNA3.1-RNASEK-b, pEGFP-RNASEK-a, and pEGFP-RNASEK-b. Cell type is indicated in each image. The relative normalized mRNA expression of genes was obtained by using the 2^−ΔΔCT^ method with β-actin as an internal control **(A–D)**. Bax/Bcl-2, the ratio of relative normalized mRNA expression of *Bax* to that of *Bcl-2*. The controls consist of pcDNA3.1-basic **(A, B, G–L)** and pEGFP-C1 **(C, D)**. All values are mean ± SD. The results are representative of three independent experiments. *P < 0.05 versus the controls. ^#^ *P* < 0.10 versus the pcDNA3.1-basic group **(L)**.

Apoptosis plays a key role in innate immunity, which allows us to think about whether RNASEK-a and -b affect apoptosis in fish. We selected CIK, CL, or CO cells as models to validate our hypothesis by using qRT-PCR, DNA ladder, TUNEL, or flow cytometry methods. In CIK cells, pcDNA3.1-RNASEK-a and -b were used to overexpress the matched genes, which separately upregulated the *Bax*/*Bcl-2* mRNA ratio by 1.45- and 1.66-fold in comparison with the controls (**Figures 5A, B**), indicating that they can enhance apoptosis. In CL cells, overexpression of RNASEK-a and -b was individually performed with pEGFP-RNASEK-a and -b transfection, increasing the *Bax*/*Bcl-2* mRNA ratio by 1.29 and 1.43 times versus the controls, respectively (**Figures 5C, D**). As for the DNA ladder in CIK (**Figure 5E**) and CL (**Figure 5F**) cells, the transfection of both pEGFP-RNASEK-a and -b produced more DNA fragmentations (indicated by red arrows), one characteristic of cell apoptosis, than the controls (**Figures 5E, F**). Besides, pcDNA3.1-RNASEK-a and -b transfection separately increased the number of apoptotic CIK cells (TUNEL-positive green signals, pointed by red arrows) by 1.93 and 2.39 times compared with the controls (**Figures 5G, H, I, J**). Furthermore, we overexpressed these two genes in CO cells *via* transfection of pcDNA3.1-RNASEK-a and -b, which separately elevated the proportion of Annexin V-positive apoptotic cells by 1.20 and 1.21 times than the control (**Figures 5K, L**). Altogether, grass carp RNASEK-a and -b individually promote cell apoptosis.

### Grass Carp RNASEK-a and -b Activate eIF2 α

**Figure 6:**
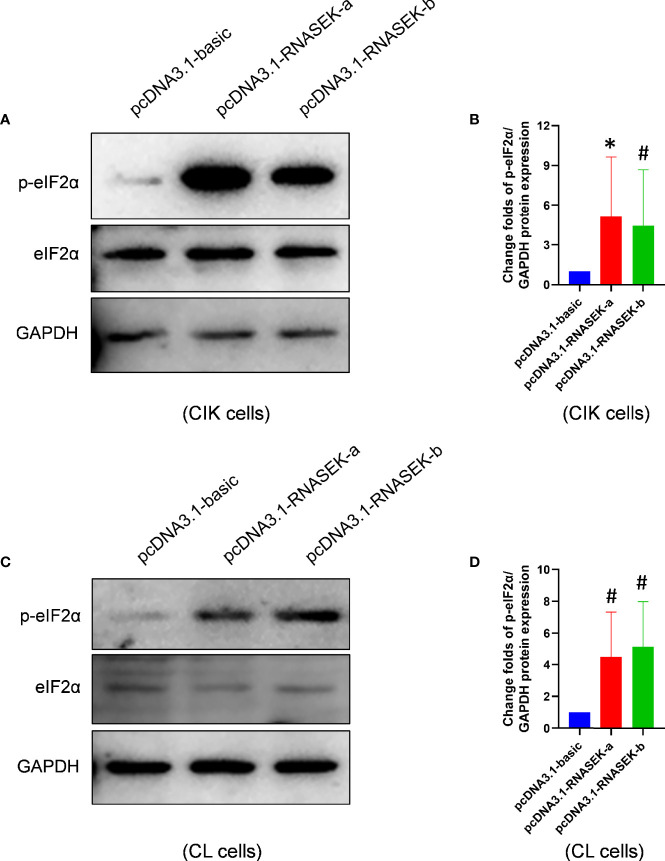
Grass carp RNASEK-a and -b individually activate eIF2α. **(A, C)** In CIK **(A)** and CL **(C)** cells, the protein levels of basal and p-eIF2α were determined by Western blot with GAPDH as a loading control. **(B, D)** The grayscale values of p-eIF2α bands representing **(A, C)** were separately calculated by the ImageJ software (*n* = 3–5; the control data were normalized to 1). Cell type is marked in each image. Overexpression of RNASEK-a and -b was conducted by transfection of pcDNA3.1-RNASEK-a and -RNASEK-b into cells for 36 h, respectively. All values are mean ± SD. These results are representative of three independent experiments. *P < 0.05 **(B)** and ^#^ *P* < 0.10 **(B, D)** versus the pcDNA3.1-basic group.

As already known, increased p-eIF2α expression promotes cell apoptosis in grass carp (30). To investigate whether the pro-apoptosis effects of both RNASEK-a and -b on apoptosis are associated with p-eIF2α, we performed overexpression experiments with transfection of pcDNA3.1-RNASEK-a and -b into CIK and CL cells. As shown in **Figure 6**, RNASEK-a and -b significantly elevated p-eIF2α protein expression by 5.14- and 4.46-fold in CIK cells as well as 4.51 and 5.14 times in CL cells compared with the controls, respectively. Taken together, grass carp RNASEK-a and -b individually activate eIF2α.

## Discussion

Whole-genome duplication (WGD) is a course of sudden doubling of the entire genome sequence, which has been widely accepted as an important mechanism for shaping vertebrate evolution (31–34). The first and second rounds of WGD took place at the origin of ancient vertebrate lineages, probably dating from about 590 and 440 million years ago (Mya), respectively (32). The third round of WGD occurred in fishes at 225–350 Mya that land vertebrates did not experience, termed as fish-specific genome duplication (FSGD) (33–36). In teleost, an excess of duplicated gene paralogs with biological function underwent FSGD. For instance, zebrafish has two paralogs of multiple important genes, including HOX clusters (e.g., *HOXB5a*/*b*, *HOXB6a*/*b*, and *HOXC6a*/*b*) which can specify cell fate in the anterior–posterior axis of embryos (31, 37) as well as *Epoa*/*b* and *Kitlga*/*b* which participate in the process of hematopoiesis (36). Christoffels et al. (38) found 468 fish-specific paralogons covering 6.6% of the Fugu genome, where two pairs of paralogs, type III receptor tyrosine kinase *cf1ra*/*b* and *pdgfrβa*/*b*, evolved as coevolutionary unit *pdgfrβ*-*csf1r* paralogons (39). In our study, the phylogenetic analysis showed that RNASEK exists in land animals but RNASEK-a and -b exist only in fish. Moreover, we searched for all species containing *RNASEK* genome from NCBI and also found that RNASEK-a and -b are fish-specific. Furthermore, the result of multiple sequence alignment (**Figure S1B**) indicated that these two proteins share about 73% of amino acid identity. Taken together, we identified *RNASEK*-*a* and -*b* as two fish-specific paralogs produced in the course of FSGD. In our genomic structure analysis (**Figure S3**), the intron and exon variation of *RNASEK*-*a* is obviously more than that of *RNASEK*-*b*, suggesting that *RNASEK*-*a* has a faster evolutionary rate than *RNASEK*-*b* in fish (39, 40). However, another question may necessitate elucidation: what is the evolutionary relationship between mammalian *RNASEK* and fish paralogs *RNASEK-a* and -*b*? There seem to be two explanations: a) fish *RNASEK-a*/*b* and mammalian *RNASEK* experienced three and two rounds of WGD, following the 1-2-4 and 1-2-4-8 rule, respectively (32, 35); b) fish *RNASEK-a* and -*b* underwent second gene loss as evolution, leading to only one form of *RNASEK* in mammals (40). Although we have provided several shreds of evidence for fish-specific paralogs *RNASEK*-*a* and -*b*, in our opinion, further investigation is necessary on their functional evolution.

As discussed above, the two paralogs RNASEK-a and -b exist only in fish, but there is a lack of reports about the comparison of their similarities or differences. In this study, we found that grass carp RNASEK-a and -b share highly similar CDS regions and domains, as well have identical sites of Cys residues (**Figure S1A**), Cys72 of which is absolutely conserved with Cys69 of human RNASEK; Cys69 is required for catalytic activity (6). Thus, RNASEK-a and -b may play similar roles in organisms and interact with each other to regulate biological processes, where conservative Cys residue-dependent catalytic activity is probably necessary. Additionally, fish RNASEK-b genome (composed of three exons; divided into only one branch) seems more stable than that of RNASEK-a (composed of three or four exons; divided into three distinct branches). Whether this evolutionary difference guides the evolution of species from fish to human remains to be clarified. Nevertheless, RNASEK-a and -b are two different functional paralogs belonging to the RNASEK family, deserving in-depth dissection to understand their functions.

Further analysis showed that grass carp RNASEK-a and -b were localized in part to endosomes required by multiple viral entries, which is in agreement with human RNASEK (9). Based on the above comparison, we investigated their expression profiles in grass carp challenged by dsRNA poly I:C and GCRV and immune-related functions. Interestingly, both RNASEK-a and -b were upregulated by the indicated stressors and enhanced type I IFN expression and apoptosis. Differently, RNASEK-a had high expression in grass carp liver, but RNASEK-b had high expression in the brain and eye. This may have two implications. Firstly, RNASEK-a is mainly involved in the immunity of the metabolic system, but RNASEK-b is involved in the neuroimmune system. Secondly, there is functional complementation between RNASEK-a and -b for maintaining the homeostasis of aquatic organisms.

Induction of type I IFNs by invading pathogens is crucial for the innate immunity of organisms (41), which is mediated by multiple PRRs, such as TLRs. Given both grass carp RNASEK-a and -b mainly localized to endosomes and endoplasmic reticulum rather than other tested organelles, we first focus our eyes on the endosomal TLRs, such as TLR3, TLR7, TLR8, TLR9, and TLR10 in mammals (42–45). TLR3 and TLR10 detect dsRNA such as poly I:C and then activate divergent downstream pathways, of which TLR3 recruits the adaptor protein TRIF and triggers the recruitment of TBK1, increasing the phosphorylation of IRF3 and IRF7 for enhancing type I IFN production (43, 44). TLR10 binds to the adaptor protein MyD88 to suppress TLR3 signaling- or IRF7-dependent type I IFN induction (45). TLR7/8 and TLR9 recognize ssRNA and unmethylated CpG DNA, respectively. They recruit the adaptor MyD88 to transmit signaling, activating IRF7 or IRF3 to increase type I IFN induction (17, 42–44).

Given the features of the subcellular localization of grass carp RNASEK-a and -b, we also pay much attention to the endosomal TLRs in fish. To date, more than 20 TLRs have been identified from different fishes, at least 10 of which localize to endosomes (17, 19). To investigate which endosomal TLRs are likely involved in the RNASEK-a/b-induced type I IFN production (**Figure 3**), we performed expression analysis of RNASEK-a and -b in grass carp and observed that both of them can be upregulated by the stimulation of poly I:C and GCRV *in vitro* and *in vivo* (**Figure 1**). Therefore, we focus on three common endosomal TLRs (TLR3, TLR19, and TLR25) that can respond to poly I:C or viruses and enhance type I IFN production. Both TLR3 and TLR19 can recognize dsRNA such as poly I:C and GCRV, then recruit the adaptor TRIF (17, 18, 46). In grass carp, the TLR3–TRIF complex activates TBK1 and further facilitates the phosphorylation of IRF3/IRF7 for enhancing type I IFN production (17, 18); the TLR19–TRIF complex elevates the expression of phosphorylated IRF3 but not TBK1 and IRF7, triggering type I IFN secretion (46). TLR25, a fish-specific TLR, can respond to LPS, bacteria, or poly I:C in *Schizothorax prenanti*, thereby activating ISRE-induced type I IFN signaling pathways (47).

In mammals, RNASEK also localizes to endosomes, assisting in the endocytosis of multiple ssRNA viruses, such as influenza A virus, rhinovirus, and flavivirus, which enter into cells through endosomal pathways (9–11). Accordingly, RNASEK may interact with endosomal TLR7/8 to activate IRF3/IRF7 to promote type I IFN secretion. Besides, we observed that RNASEK-a and -b could respond to dsRNA poly I:C and GCRV in grass carp, suggesting they are probably involved in fish endosomal TLR-dependent signaling.

In grass carp, endosomal TLR3, TLR7/8, TLR9, TLR19, and TLR25 from above were identified, which facilitate the phosphorylation of either IRF3 or IRF7 to induce type I IFN production (17). These analyses raise a question: do grass carp RNASEK-a and -b promote type I IFN production through endosomal TLR-dependent pathways? Fortunately, we found that both of them positively regulated type I IFN at the transcriptional and protein levels in CIK or CO cells (**Figure 3**). Furthermore, RNASEK-a and -b individually upregulated the phosphorylation of IRF3 and IRF7 in CIK cells (**Figure 4**). Thus, we postulate that both of them induce type I IFN *via* interacting with endosomal TLR–IRF3/IRF7 signaling axis, which needs to be confirmed in the future. As already known, the endosomal pathway has been essential for the internalization of a large number of viruses with dsRNA and ssRNA genome, such as GCRV, Zika virus, and SARS-CoV-2 (48–51). Among them, SARS-CoV-2 can be restricted by LY6E, one protein induced by IFN and downstream of RNASEK that helps viral entry (11, 51). But on the other hand, LY6E facilitates viral entry from endosomal compartments or replication, such as the West Nile virus and HIV-1 (52, 53). Likewise, RNASEK assists in viral uptake and, at the same time, promotes type I IFN production (**Figure 3**), which may be reasonable and necessary to maintain the homeostasis of host defense against pathogen invasion. Altogether, RNASEK plays a pivotal role in multiple serious viral infections; hence, detailed molecular mechanism by which RNASEK enhances type I IFN secretion for antiviral response should be elucidated as soon as possible.

Apoptosis is involved in various biological processes, such as viral infections and cancers (54, 55). Paclitaxel, an anti-cancer drug, induces the apoptosis of BT-20 breast and SKOV-3 ovarian cancer cells, accompanying the elevated expression of RNASEK (up to 9-fold) (7). Thus, is RNASEK related to apoptosis-related pathways? Expectedly, we found that both grass carp RNASEK-a and -b promoted apoptosis in different cell models. In the knockdown experiments, we exclusively observed the decreased alterations of *Bax*/*Bcl-2* mRNA ratio, an indicator for cell apoptosis (56), but there were no changes in other assays (data not shown), which may be because apoptosis is opt to be affected by higher but not lower expression of RNASEK. Furthermore, paclitaxel induces endoplasmic reticulum stress-related apoptosis of SK-N-SH neuroblastoma cells in part *via* increasing eIF2α phosphorylation (55). Accordingly, does RNASEK promote apoptosis through activating eIF2α? Indeed, overexpression of both grass carp RNASEK-a and -b upregulated the phosphorylation of eIF2α in CIK and CL cells as expected. Additionally, PERK, PKR, and PKZ all reduce cell viability or promote cell apoptosis *via* activating eIF2α in grass carp (30, 57, 58). Therefore, PERK and PKR/PKZ may be involved in RNASEK-a/b–eIF2α axis-induced apoptosis in fish. Moreover, a bridge between IFN and apoptosis has been established in host defense against viral infections (54). Hence, given that the pro-apoptosis effects of RNASEK are associated with various cancers or other immune-related diseases, clarifying the mechanism by which RNASEK exerts its function is much necessary.

Our study has several limitations. Firstly, the enhanced effects of RNASEK on type I IFN production and apoptosis were confirmed only in grass carp cells because of the restricted experimental conditions, which may need to be further validated in gene-edited fish or mammals. Additionally, basal protein expression of RNASEK-a, RNASEK-b, Bax, and Bcl-2 in cells was not detected due to the lack of specific antibodies against each of them in grass carp. Instead, we tested the mRNA alterations of these genes in different grass carp cells. Moreover, we found that type I IFN protein bands were very weak when observed through Western blot in CIK cells because of the lower potency of anti-type I IFN antibody. Alternatively, we tested type I IFN protein levels by using immunofluorescent staining in CIK cells.

In conclusion, we demonstrate for the first time that RNASEK enhances type I IFN secretion and promotes apoptosis. We first cloned the two paralogs RNASEK-a and -b in grass carp which has nearly identical innate immune-related pathways with those of mammals (13, 18). Grass carp RNASEK-a and -b have the same size of protein with two transmembrane domains and a highly similar amino acid sequence but possess their unique features. In addition, RNASEK-a and -b localized in the cytoplasm and had colocalization with each other. Fine subcellular localization showed that they localized to early and late endosomes and endoplasmic reticulum but rarely to the mitochondria and lysosomes. Intriguingly, dsRNA poly I:C and GCRV can upregulate RNASEK-a and -b *in vivo* and *in vitro*. Furthermore, we found that RNASEK-a and -b not only induced type I IFN production through activating IRF3/IRF7 but also enhanced apoptosis *via* activating eIF2α in multiple grass carp cells. Taken together, our study reveals a previously unrecognized role of RNASEK as a novel positive regulator of type I IFN secretion and apoptosis, hence providing new insights into understanding the mechanism by which RNASEK regulates multiple pathological processes.

## Data Availability Statement

The datasets presented in this study can be found in online repositories. The names of the repository/repositories and accession number(s) can be found in the article/**Supplementary Material**.

## Ethics Statement

The animal study was reviewed and approved by Nanchang University.

## Author Contributions

CH and XLT supervised the research. ZCS conceived the study and designed and performed the experiments. ZJ, XX, and ML analyzed the experimental data. QZ, YZ, YL, and SW provided reagents and technical assistance and contributed to completion of the study. ZCS wrote the manuscript. All authors contributed to the article and approved the submitted version.

## Funding

This study was supported by research grants from major projects of the Natural Science Foundation of Jiangxi Province (20171ACB20004); the National Natural Science Foundation of China (31960735); the earmarked fund for Jiangxi Agriculture Research System (JXARS-06); the National Key Research and Development Program of the Ministry of Science and Technology, China (2020YFC2002900 to XLT); the Key Program of the National Natural Science Foundation of China (81630034 to XLT); and the Key Programs (20192ACB70002, 20181ACB20017, and 20181BCD40001) of Jiangxi Province, China.

## Conflict of Interest

The authors declare that the research was conducted in the absence of any commercial or financial relationships that could be construed as a potential conflict of interest.

## Publisher’s Note

All claims expressed in this article are solely those of the authors and do not necessarily represent those of their affiliated organizations, or those of the publisher, the editors and the reviewers. Any product that may be evaluated in this article, or claim that may be made by its manufacturer, is not guaranteed or endorsed by the publisher.
