# Supplementary material for: Fish Paralog Proteins RNASEK-a and -b Enhance Type I Interferon Secretion and Promote Apoptosis
Source: Front Immunol. 2021 Nov 22;12:762162. doi: 10.3389/fimmu.2021.762162 (PMC8645942; doi:10.3389/fimmu.2021.762162)
Supplement: Supplementary file 1 [file DataSheet_1.docx]

Table S1. Sequences and application of primers, siRNA, and shRNA used in this study

| Primer name | Sequence (5'-3') | Application |
| --- | --- | --- |
| RNASEK-a-ORF-F | ATGGCTTCTTTGCTTTTCTGC | CDS and genome cloning |
| RNASEK-a-ORF-R | TTAATGCACCAGATATTCCTT |  |
| RNASEK-b-ORF-F | ATGCCGTCCCTTTTATTCTGT |  |
| RNASEK-b-ORF-R | TTATGTGACCATGTACTCCTG |  |
| RNASEK-a-pcDNA3.1-Hind III-F | CCCAAGCTTATGGCTTCTTTGCTTTTCTGC | Prokaryotic/eukaryotic |
| RNASEK-a-pcDNA3.1-EcoR I-R | CCGGAATTCTTAATGCACCAGATATTCCTT | expression vector |
| RNASEK-b-pcDNA3.1-Hind III-F | CCCAAGCTTATGCCGTCCCTTTTATTCTGT | construction |
| RNASEK-b-pcDNA3.1-EcoR I-R | CCGGAATTCTTATGTGACCATGTACTCCTG |  |
| RNASEK-a-GFP-hind III-F | CCCAAGCTTCGATGGCTTCTTTGCTTTTCTGC |  |
| RNASEK-a-GFP-EcoR I-R | CCGGAATTCTTAATGCACCAGATATTCCTT |  |
| RNASEK-b-GFP-Hind III-F | CCCAAGCTTCGATGCCGTCCCTTTTATTCTGT |  |
| RNASEK-b-GFP-EcoR I-R | CCGGAATTCTTATGTGACCATGTACTCCTG |  |
| RNASEK-a-FLAG-Hind III-F | CCCAAGCTTATGGCTTCTTTGCTTTTCTGC |  |
| RNASEK-a-FLAG-EcoR I-R | CCGGAATTCATTAATGCACCAGATATTCCTT |  |
| RNASEK-b-FLAG-Hind III-F | CCCAAGCTTATGCCGTCCCTTTTATTCTGT |  |
| RNASEK-b-FLAG-EcoR I-R | CCGGAATTCATTATGTGACCATGTACTCCTG |  |
| RNASEK-a-RT-F | ATGGCTTCTTTGCTTTTCTGC | Quantitative Real-time PCR |
| RNASEK-a-RT-R | AAATGATAGAAATCCTATGCC |  |
| RNASEK-b-RT-F | ATGCCGTCCCTTTTATTCTGT |  |
| RNASEK-b-RT-R | AGAGACACTGCGCCAACACCT |  |
| IFN1-RT-F | AGAGCACATGAATTCAGTGCA |  |
| IFN1-RT-R | ATGTTTCACAGCTCTCCGGAT |  |
| Bcl-2-RT-F | GACTCCTCTCCAAACTCTGAC |  |
| Bcl-2-RT-R | TCCTTTCTATCTCGTCTCCAG |  |
| Bax-RT-F | CTCATCAGGGTGGTAAGACAT |  |
| Bax-RT-R | CCTATCACCAATCACTTTAATG |  |
| β-actin-F | CACTGTGCCCATCTACGA |  |
| β-actin-R | CCATCTCCTGCTCGAAGTC |  |
| NC | UUCUCCGAACGUGUCACGUTT | Knockdown |
| siRNASEK-a-22 | GGUCCUAAACUGGCAGCAUTT |  |
| siRNASEK-a-101 | CCACACAUUCAGCUGUGCUTT |  |
| GP-shNC | TTCTCCGAACGTGTCACGT |  |
| GP-shRNASEK-b-22 | GGGCCCAAATTGGCAGCCTGT |  |
| shNC | GGTTCTCCGAACGTGTCACGT |  |
| shRNASEK-b-120 | GATCGAAGATGTTCCCTTTAC |  |
| shRNASEK-b-226 | GCTGCCATCTACGTAGGTGTT |  |

Table S2. GenBank accession numbers of selected RNASEK orthologs from various species.

| Species | Gene | Gene ID | Protein ID |
| --- | --- | --- | --- |
| *Homo sapiens* | *RNASEK* | 440400 | NP_001004333 |
| *Macaca fascicularis* | *RNASEK* | 101866043 | NP_001270430 |
| *Macaca mulatta* | *RNASEK* | 100423494 | NP_001181733 |
| *Mus musculus* | *RNASEK* | 52898 | NP_776103 |
| *Rattus norvegicus* | *RNASEK* | 287453 | NP_001131033 |
| *Bos taurus* | *RNASEK* | 512886 | NP_001029601 |
| *Artibeus jamaicensis* | *RNASEK* | 119037170 | XP_036985131 |
| *Sturnira hondurensis* | *RNASEK* | 118979405 | XP_036891335 |
| *Gallus gallus* | *RNASEK* | 107051485 | XP_025001734 |
| *Phasianus colchicus* | *RNASEK* | 116239354 | XP_031465305 |
| *Thamnophis elegans* | *RNASEK* | 116522962 | XP_032093980 |
| *Dermochelys coriacea* | *RNASEK* | 119849341 | XP_038242208 |
| *Chelonoidis abingdonii* | *RNASEK* | 116822568 | XP_032632442 |
| *Xenopus tropicalis* | *RNASEK* | 100493134 | XP_002940100 |
| *Danio rerio* | *RNASEK-a* | 751692 | NP_001038870 |
| *Sinocyclocheilus rhinocerous* | *RNASEK-a* | 107750577 | XP_016421523 |
| *Ctenopharyngodon idella* | *RNASEK-a* | MW526956 | MW478713 |
| *Seriola dumerili* | *RNASEK-a* | 111223570 | XP_022603484 |
| *Monopterus albus* | *RNASEK-a* | 109957263 | XP_020450656 |
| *Micropterus salmoides* | *RNASEK-a* | 119912248 | XP_038587270 |
| *Danio rerio* | *RNASEK-b* | 336290 | NP_001038840 |
| *Sinocyclocheilus rhinocerous* | *RNASEK-b* | 107720683 | XP_016383955 |
| *Ctenopharyngodon idella* | *RNASEK-b* | MW526957 | MW478714 |
| *Seriola dumerili* | *RNASEK-b* | 111221370 | XP_022600453 |
| *Monopterus albus* | *RNASEK-b* | 109952256 | XP_020442897 |
| *Micropterus salmoides* | *RNASEK-b* | 119895655 | XP_038564836 |
| *Drosophila melanogaster* | *RNASEK* | 3355016 | NP_001260699 |
| *Petromyzon marinus* | *RNASEK* | 116958270 | XP_032836693 |


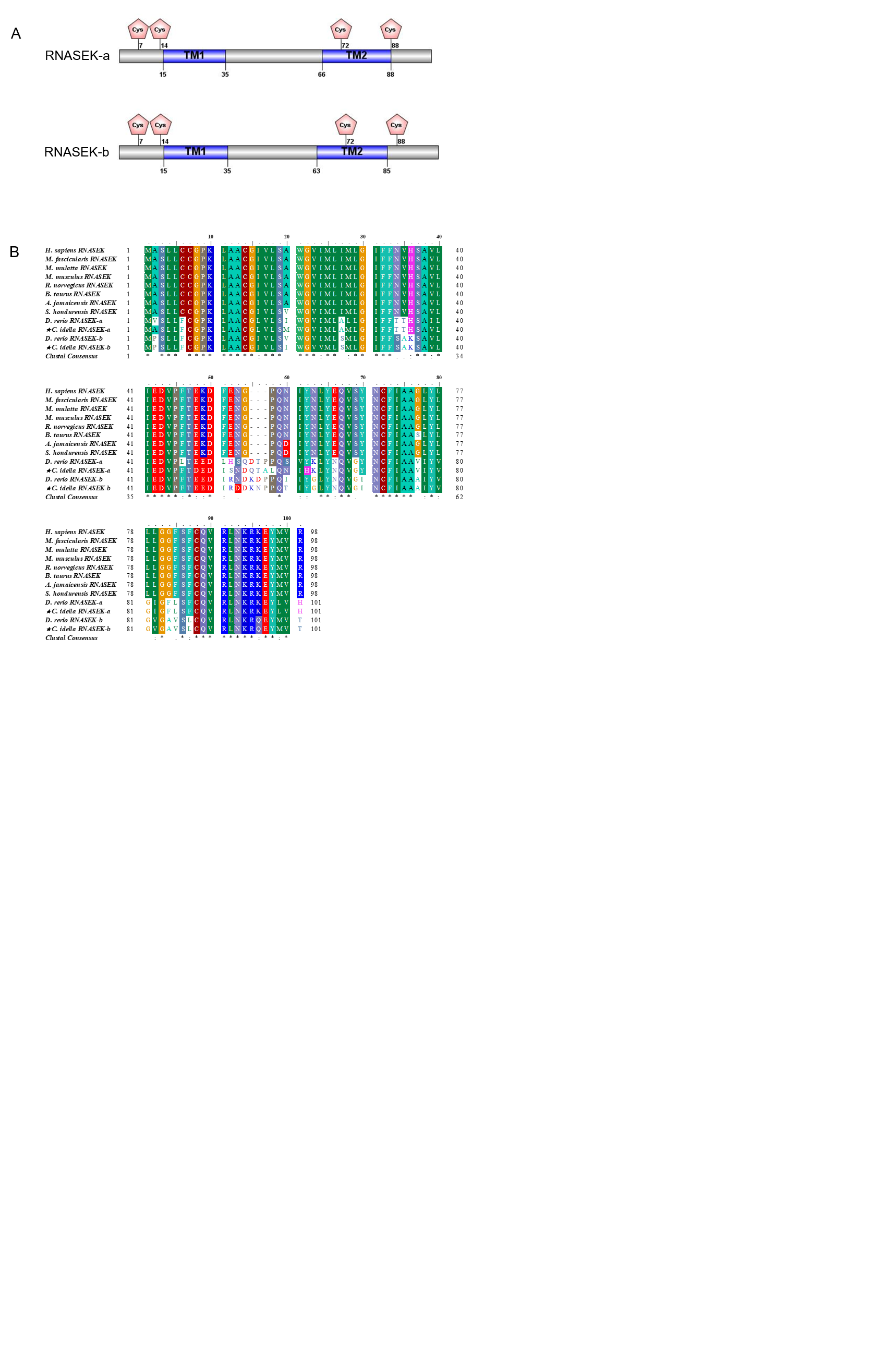


Figure S1. CDS analysis of grass carp RNASEK-a and -b. A, Domains of both RNASEK-a and -b proteins were predicted by SAMART program and Cys residues are marked, where numbers indicate the sites of relative amino acid residues. TM, transmembrane. B, Multiple sequence alignment of RNASEK orthologs was conducted by BioEdit software according to their amino acid sequences. The GenBank accession numbers of selected RNASEK orthologs from different animals are listed in Table S2. RNASEK-a and -b are two paralog proteins of RNASEK family. *Ctenopharyngodon idella* RNASEK-a and -b are indicated by the symbol ★.

.


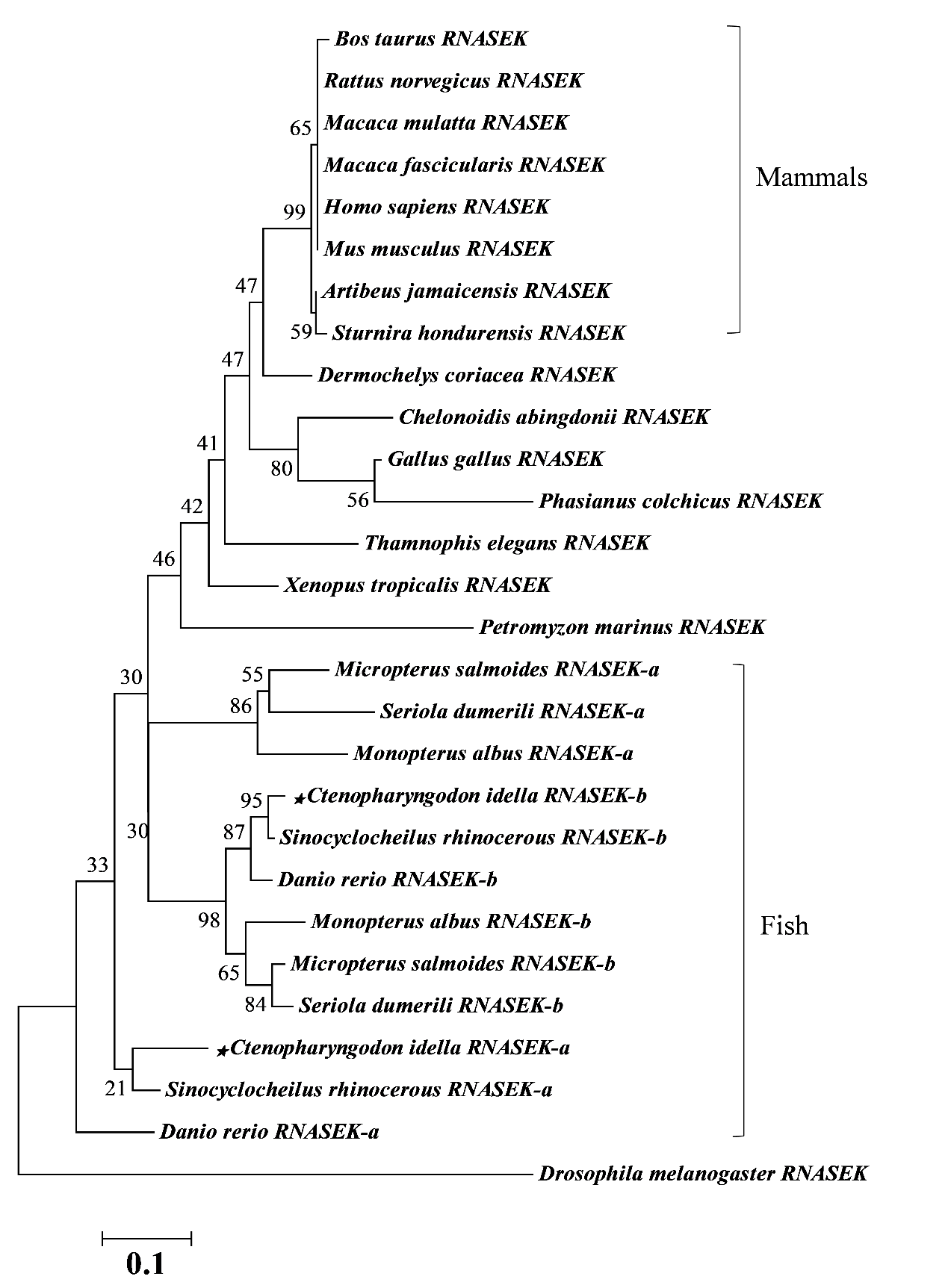


Figure S2. Phylogenetic tree of RNASEK among various species. The phylogenetic tree was conducted by using the Neighbor-Joining algorithm of MEGA6.0 software according to the amino acid sequences of RNASEK orthologs. The GenBank accession numbers of selected RNASEK orthologs from different species are listed in Table S2. RNASEK-a and -b are two paralog proteins of RNASEK family. *Ctenopharyngodon idella* RNASEK-a and -b are indicated by the symbol ★.


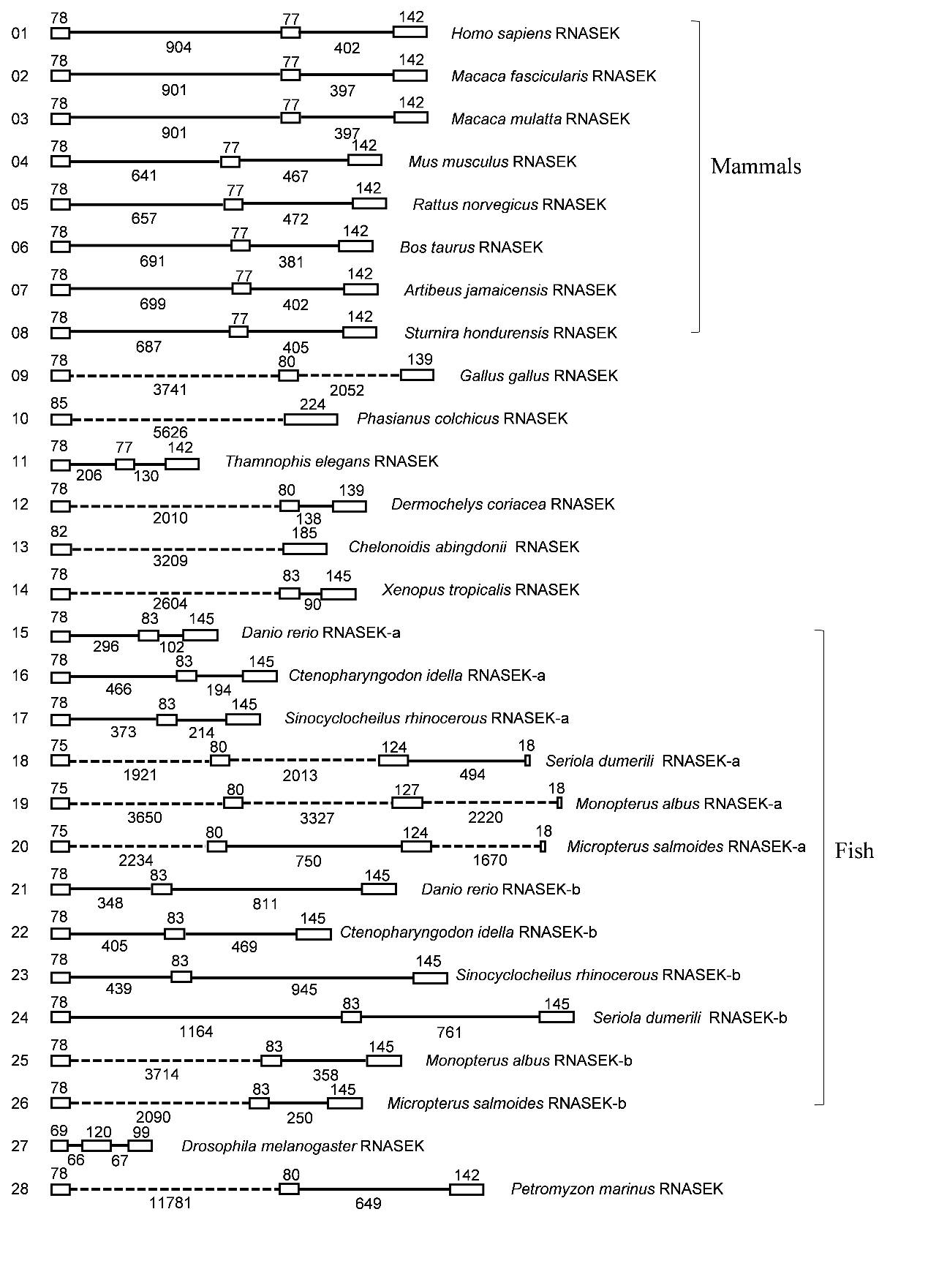


Figure S3. Genomic structure of *RNASEK* among various species. The 5’- and 3’- ends of these homologous genes are on the left and right, respectively. In the genomes of *RNASEK* orthologs, exons are represented with blank rectangles and introns with horizontal lines. The numbers above the boxes mark the size of exons, and those under lines indicate the size of introns (introns longer than 1500 bp are indicated by dashed lines). All genomic sequences source from NCBI. The GenBank accession numbers of these genes are listed in Table S2. *RNASEK-a* and *-b* are two paralogs of *RNASEK* family. Each item is described as latin name of the specie plus the gene symbol.


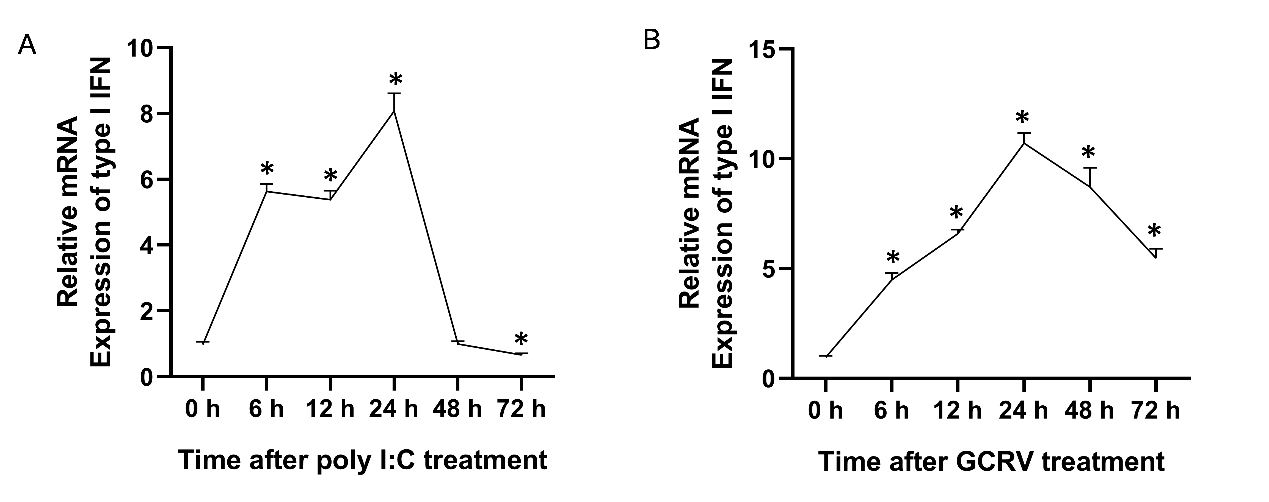


Figure S4. Expression profiles of type Ι IFN in CIK cells exposed to poly I:C (A) and GCRV (B) for multiple time periods. Templates used are the same as those in Figure 3. mRNA expression was quantified by qRT-PCR in triplicate with β-actin as an internal control. All values are mean ± S.D.. The results are representative of three independent experiments. **P* < 0.05 versus 0 h for each group.


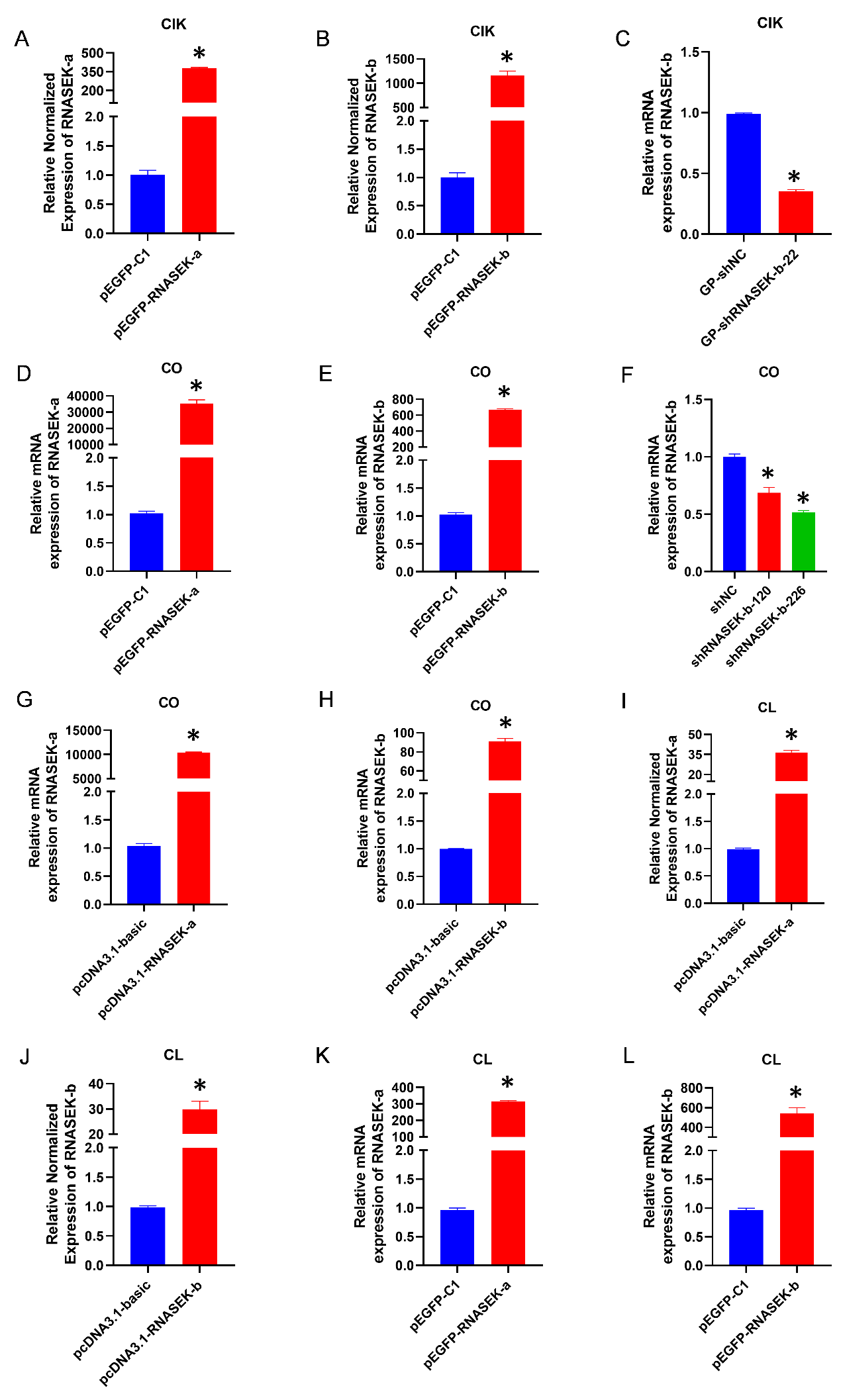
Figure S5. Overexpression and knockdown of *RNASEK-a* and *-b* were available in CIK, CO, or CL cells. Overexpression experiments were performed with transfection of different recombinant plasmids, including pEGFP-RNASEK-a (A, D, and K), pEGFP-RNASEK-b (B, E, and L), pcDNA3.1-RNASEK-a (G and I), and pcDNA3.1-RNASEK-b (H and J). Knockdown experiments were conducted by transfection of siRNA or shRNA against target genes, including GP-shRNASEK-b-22 (C), shRNASEK-b-120, and -226 (F). mRNA expression was quantified by qRT-PCR in triplicate with β-actin as an internal control. All values are mean ± S.D.. Cell types used are annotated above each figure. The results are representative of three independent experiments. **P* < 0.05 versus the controls, including pEGFP-C1 (A, B, D, E, K, and L), pcDNA3.1-basic (G-J), GP-shNC (C), and shNC (F).


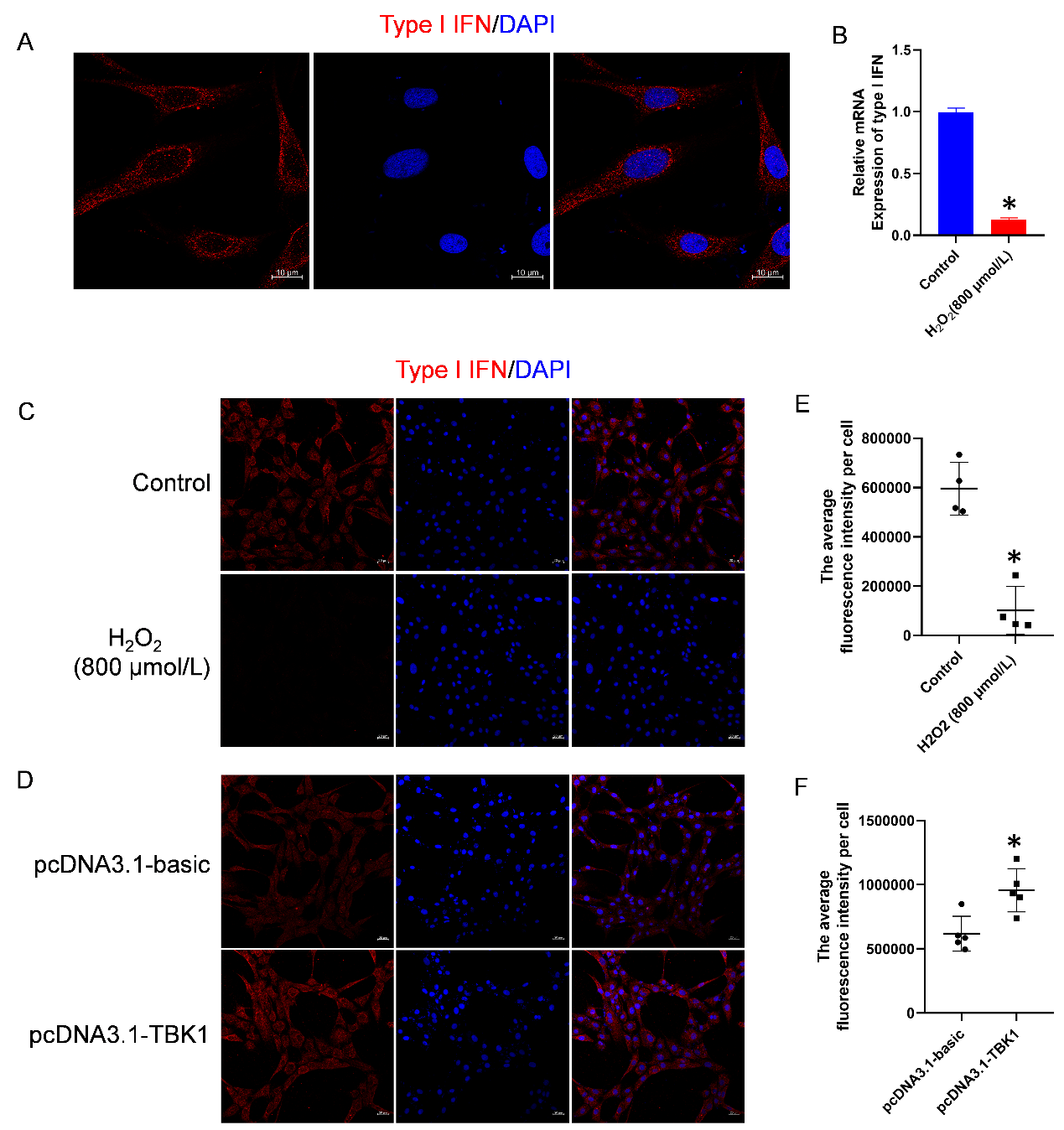


Figure S6. The specific antibody against grass carp type Ι IFN was available in CIK cells. A, The fluorescence signals of type Ι IFN existed in the cytoplasm. Images were taken under 63 × oil immersion objective lens (630 × magnification) of Zeiss confocal microscope (red, type Ι IFN; blue, DAPI; scale bar, 10 μm). B-C, There was low expression of type Ι IFN at transcriptional (B) and protein (C) levels in CIK cells after exposure to 800 μmol/L H_2_O_2_ for 4 h. D, higher expression of type Ι IFN protein was observed in CIK cells after overexpression of TBK1, one protein promoting type Ι IFN production. Images in C and D were captured under 200 × magnification using Zeiss confocal microscope (red, type Ι IFN; blue, DAPI; scale bar, 20 μm). E-F, Five views representing C and D were randomly selected to calculate statistical significance, respectively. mRNA expression was detected by qRT-PCR in triplicate with β-actin as an internal control. Type Ι IFN protein expression was determined by using immunofluorescent staining with specific antibody. All values are mean ± S.D.. The results are representative of three independent experiments. **P* < 0.05 versus the control (B, C, and E) and pcDNA3.1-basic (D and F) groups.


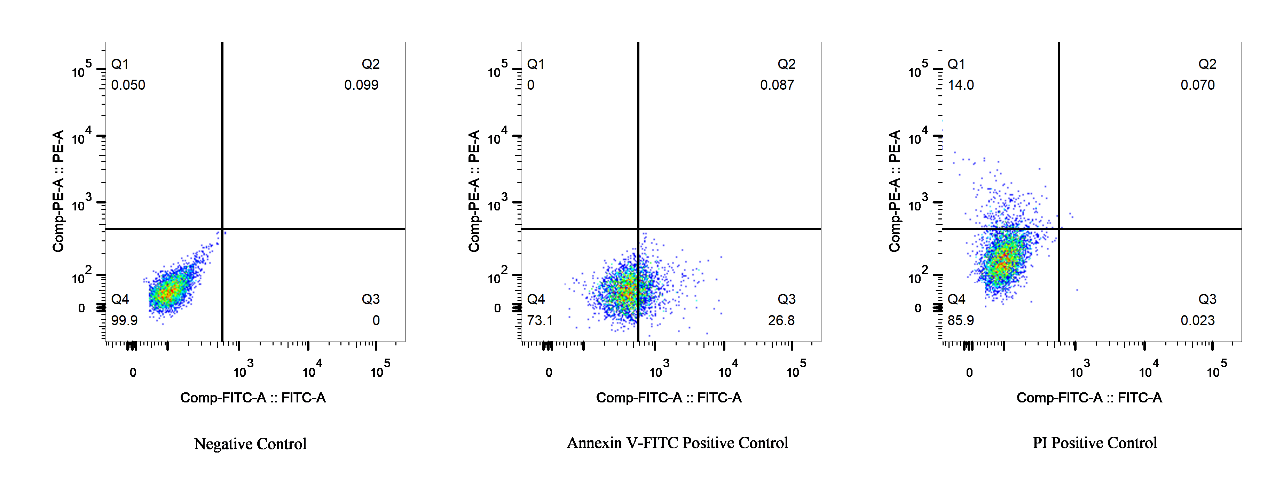


Figure S7. The controls of flow cytometry used to test CO cell apoptosis were available for Figure 5J. Negative control has no signals in Annexin Ⅴ-FITC or PI positive regions. Annexin Ⅴ-FITC or PI single staining have no signals in PI or Annexin Ⅴ-FITC positive regions, respectively. The results are representative of three independent experiments.
